# On the Means of Lessening the Pains and Danger of Child-Bearing, and of Preventing Its Consequent Diseases; in a Letter to Dr. Edward Miller

**Published:** 1808-07

**Authors:** Benjamin Rush

**Affiliations:** Professor of Medicine in the University of Pennsylvania


### *On the Means of lessening the* Pains *and* Danger *of* Child-Bearing, *and of preventing its consequent Diseases; in a Letter to Dr.* Edward Miller, *by* Benjamin Rush, *M. D. Professor of Medicine in the University of Pennsylvania.*

Dear Sir,

It has generally been supposed, that a necessary and unchangeable connection exists between pain and child-bearing. Such a connection, it is true, was established between them, as part of the curse inflicted upon woman after the loss of her primeval innocence. A similar connection was established between labour and the sweat of the brow in man; and yet thousands of our fellow men live without labour, in the enjoyment of all the comforts of life. Even the curse which was inflicted upon the earth has been in part repealed, by the spontaneous or artificial fruitfulness which has succeeded to briars and thorns in many countries. The mitigation of the pains connected with child-bearing has been effected in Turkey, by the use of sweet oil (which acts only as a purge) during the last months of pregnancy. A similar alleviation of those pains has been produced by climate in a part of Africa, in the Brazils, in Callabria, in Sicily, and in several of the West India islands. A scanty diet has so far lessened them in the Indian women of our country, that they are able to work a few hours after their delivery, which is sometimes effected without the aid of a human creature. Heaven, in these instances, has kindly intimated to us, that the pains of child-bearing are not entailed upon the female sex by an immutable law. In our attempts, therefore, to improve and extend the means of lessening them, we do but develope the kind and benevolent disposition of the Creator of the world to the human race.

To induce a belief in the efficacy of certain remedies which are calculated to lessen, and, perhaps, finally to destroy, the pains of child-bearing at all times, and in all states of society, it will be necessary to premise the following propositions.

1. Previously to child-bearing, the female system is generally plethoric, and unduly excitable, from the ratio between exercise and the appetite being destroyed by an excess of the latter during pregnancy, and from the influence of the distended state of the uterus upon the whole system.

 2. Child-

2. Child-bearing is a disease. The form of this disease is a spasm of a clonic nature. It does not militate against the truth of this proposition, that the morbid affection of the womb is not attended with inflammation; for we now know that disease does not consist in inflammation, that it is one of its effects only, and that the highest grade of disease often exists without inflammation. The symptoms of this parturient disease sometimes appear in chills, preternatural heat, thirst, a quick, frequent, full, and tense pulse, pains in the head and other parts of the body.

3. The operations of Nature in this disease, as in most others, are excessive, deficient, or not properly directed to accomplish her purposes. Their *excess* appears occasionally in great pains in the bowels, in hæmorrhages from the uterus, in convulsions of the whole nervous system, in the total absence of contraction in the uterus from the suffocation of its morbid excitement, in the wrong position of the fœtus in utero, and in the protraction of exquisite and fruitless pains far beyond the time necessary to exclude the fœtus. The operations of nature in child-bearing, I believe, are not often deficient in force. Women who have been debilitated by previous diseases, or by accidental evacuations of any kind, have generally very easy and natual labours.

As a natural inference from the above propositions, I go on to remark, that the remedies for difficult and painful parturition should be the same as for all other convulsive and spasmodic diseases, taking care to vary them according to the force of the disease and the state of the system. As the difficulty and extreme pain of child-bearing arise chiefly from an excess of natural force, or from an improper direction of it, I shall first mention the remedies for lessening its pain and shortening its duration.

The first of these remedies is blood-letting. I suggested the use of this remedy, with most of the above reasons in its favour, in my Lectures, in the year 1791. On the 11th of January, 1795, a man came into the Pennsylvania Hospital, whose shoulder-bone had been luxated for two months. All the usual methods of reducing it had been tried to no purpose. Dr. Physick, to whose care he was committed, began his preparations to relieve him, by drawing blood from his arm until he fainted. While he lay in this insensible state, the Doctor reduced the bone with but little force, and nearly in the twinkling of an eye. This easy and happy cure increased my confidence in the lancet in parturition. It was confirmed still more by recollecting its its controuling influence over the morbid excitement of the uterus, in preventing abortions in the yellow fever of 1793, and by reflecting on the instant relief which it affords in colics, convulsions, and all other violent diseases, which affect, at the same time, the muscular and arterial systems. But I am happy in being able to add, that my opinion of the efficacy of blood-letting in abating the pains and danger of child-bearing, no longer rests upon reasoning and analogy. Dr. Dewees, of this city, has adopted it with the happiest effects. Dr. Brown, formerly a pupil of Dr. Physick, has lately used it in his practice in Chester county, in our State, to an extent which has added greatly to the benefits derived from it. He seldom draws less than thirty ounces of blood in the beginning of a labour. The consequences of this copious loss of blood, in the diminution of pain, and accelerating the exclusion of the fœtus, have been highly grateful to his patients, and pleasing to their friends. Considering the predisposing causes of plethora and excitability which have been mentioned, I am disposed to believe a much larger quantity of blood than thirty ounces might be drawn in some cases, and at certain times, with advantage. These times are in the winter and spring months, and during the prevalence of inflammatory and malignant epidemics; for, in the latter period, Hippocrates long ago observed labours to be more difficult than at any other time.* It is from the influence of the same state of atmosphere which produces these more difficult labours, that abortions are so common during the prevalence of the plague in all countries.†

I am aware that this remedy is not a new one to a certain degree; but I believe it has never yet been advised nor used in the copious manner, nor regulated by the morbid phenomena of child-bearing, which have been mentioned.

2. In addition to the loss of blood, suited in quantity to the symptoms of the parturient disease, the bowels should be plentifully opened by means of glysters, and the diet and drinks should contain as little stimulus and nourishment as possible.

3. Perhaps an advantage would arise from advising a low diet to pregnant women for ten days or two weeks before they are confined, also from the use of lenient purges once or twice a week, agreeably to the Turkish practice, during the

† Diemerbroeck de Peste.

the three last months of their pregnancy. The good effects of this regimen in lessening the violence of the small-pox and yellow fever, encourage us to expect similar benefits from it in the violent disease of parturition.

I have hitherto advised the use of blood-letting, and other depleting remedies, only as the means of lessening the pains and danger of child-bearing; but their advantages would probably extend much further by preventing the after-pains—hæmorrhages—obstructed lochia—retention of the placenta—prolapsus uteri—inflamed breasts—swelled legs—colics—madness and fevers, which so often follow delivery. Even the fœtus would probably be benefited by the use of the above remedies, by suffering less from pressure and bruises, in a tedious and painful passage into the world.

You will perceive, from what has been advanced, that the principle design of this communication is to suggest that a morbid action may be converted into a natural one, and thereby placed upon a footing with other actions of the body which are painful or tedious only from disease. The lower limbs and the arms, which suffer exquisitely in a rheumatism, are overstretched, without pain, in running, and lifting heavy weights. The blood-vessels, likewise, when excited into morbid action in a fever, communicate pain to the head and other parts of the body; but the same vessels, when increased in their force and dimensions by the equable and healthy action induced by violent exercise, are never known to produce pain, and subside by rest, without danger or injury to any part of the system.

The cases of difficult and painful delivery, from a state of the system below that to which depleting remedies are calculated to reduce it, I have hinted, rarely occur. Most of the labours I have met with, or heard of, in the last stage of chronic diseases, have been short, and comparatively easy.

Where there is reason to believe the contractions in the uterus will be too weak to expel the fœtus, a cordial diet should be advised previously to the expected time of confinement; and where the absence of throes, and the slow progress of delivery, indicate a deficiency of muscular irritability in the fibres of the uterus, opium should be given in proportion to the exigences of the system. I think I have seen the happiest effects from that excellent medicine, in such cases, in destroying useless pains, and shortening the duration of labour. How far a medicine so powerful (if such can be found) as wholly to suspend the sensibillity of of the nerves, without impairing their irritability, might succeed in destroying pain altogether, I know not; but the following fact shows that it has been destroyed by a disease which produced that effect upon the nerves. A lady, who had been for several years subject to epilepsy, was delivered of a child in one of her fits. Immediately afterwards she fell asleep. Her child was dressed, and laid by her side. In the morning, as soon as she awoke, she saw this child, and asked with surprise whose it was. Upon being told it was hers, she declared that she had no recollection of having felt the least pain in bringing it into the world. This information was communicated to me by the husband of the lady, a few hours after her delivery, for I was then attending her for another indisposition. In this instance we see what Dr. Darwin calls a sensitive and irritative disease, converted into a disease wholly irritative.

*Philadelphia, May* 12, 1802.

*P. S.* In looking over some old letters upon medical subjects, I met with a communication from one of my former pupils, Dr. Bullus, which forcibly illustrates the principles and practice contained in the foregoing letter upon the parturient disease. In the month of July, 1797, the Doctor was called upon at Reading, in Pennsylvania, to attend a women, about the last month of her pregnancy, in a malignant fever. By the use of copious bleeding and a salivation she recovered. But her labour soon afterwards came on, "and (says the Doctor), as it continued rather long, I bled her twice. Immediately after binding up her arm, I delivered her of two fine boys, without any difficulty to myself and with scarcely any pain to my poor patient. She has since told me that she had never experienced so little pain in any of her former labours, which have been fourteen in number, and that if she were to undergo twenty more she would always be bled in them." In a subsequent letter, dated July 30th of the same year, the Doctor says, his patient, whose name is Ann Harkner, had consented to the publication of her case, and that, if necessary, she would subscribe it with her name.

CRITICAL

